# Polybrominated Diphenyl Ether Levels in the Blood of Pregnant Women Living in an Agricultural Community in California

**DOI:** 10.1289/ehp.8899

**Published:** 2006-10-19

**Authors:** Asa Bradman, Laura Fenster, Andreas Sjödin, Richard S. Jones, Donald G. Patterson, Brenda Eskenazi

**Affiliations:** 1 Center for Children’s Environmental Health Research, School of Public Health, University of California, Berkeley, California, USA; 2 Division of Environmental and Occupational Disease Control, California Department of Health Services, Oakland, California, USA; 3 Division of Laboratory Sciences, National Center for Environmental Health, Centers for Disease Control and Prevention, Atlanta, Georgia, USA

**Keywords:** blood, brominated flame retardant, exposure, Mexican, PBDE, polybrominated diphenyl ethers, pregnant

## Abstract

**Background:**

Recent studies have raised concerns about polybrominated diphenyl ether (PBDE) flame retardant exposures to pregnant women and women of child-bearing age in the United States. Few studies have measured PBDEs in immigrant populations.

**Objectives:**

Our goal was to characterize levels of seven PBDE congeners, polychlorinated biphenyl (PCB)-153, and polybrominated biphenyl (PBB)-153 in plasma from 24 pregnant women of Mexican descent living in an agricultural community in California.

**Results:**

The median concentration of the sum of the PBDE congeners was 21 ng/g lipid and ranged from 5.3 to 320 ng/g lipid. Consistent with other studies, 2,2′,4,4′-tetrabromodiphenyl ether (BDE-47) was found at the highest concentration (median = 11 ng/g lipid; range, 2.5–205) followed by 2,2′,4,4′,5-pentabromobiphenyl (BDE-99) (median = 2.9 ng/g lipid; range, 0.5–54), 2,2′,4,4′,5-pentaBDE (BDE-100) (median = 1.8 ng/g lipid; range, 0.6–44), and 2,2′,4,4′,5,5′-hexaBDE (BDE-153) (median = 1.5 ng/g lipid; range, 0.4–35). Levels of PCB-153 (median= 4.4 ng/g lipid; range, < 2–75) were lower than U.S. averages and uncorrelated with PBDE levels, suggesting different exposure routes.

**Conclusions:**

The overall levels of PBDEs found were lower than levels observed in other U.S. populations, although still higher than those observed previously in Europe or Japan. The upper range of exposure is similar to what has been reported in other U.S. populations. PBDEs have been associated with adverse developmental effects in animals. Future studies are needed to determine the sources and pathways of PBDE exposures and whether these exposures have adverse effects on human health.

Recent reports have suggested that women of reproductive age in the United States may have a higher exposure to flame retardants, particularly polybrominated diphenyl ethers (PBDEs) (Petreas et al. 2003; Schecter et al. 2003; She et al. 2002), than their counterparts in European countries (Guvenius et al. 2003; Sjödin et al. 1999, 2001b; Thomsen et al. 2001). In addition, studies from the United States have shown that PBDE levels have increased in recent decades (Petreas et al. 2003; Sjödin et al. 2004b). Some evidence suggests that these chemicals have the potential to affect neurodevelopment and the endocrine system in animals (Birnbaum and Staskal 2004; Legler and Brouwer 2003). The route of exposure in humans is thought to be through food, ingestion of dust (Schecter et al. 2004; Sjödin et al. 2004b, 2005), and by inhalation (Sjödin et al. 1999, 2001a), but further study is required to elucidate these pathways.

In the present study we report the results of PBDE measurements in a pilot study of immigrant Mexican pregnant women.

## Materials and Methods

We selected a convenience sample of 24 women who participated in the Center for the Health Assessment of Mothers and Children of Salinas (CHAMACOS) project, a longitudinal birth cohort study of 601 pregnant women living in the Salinas Valley, California (Eskenazi et al. 2003). To be eligible, a women had to be enrolled in MediCal, > 18 years of age, < 20 weeks gestation at enrollment, and speak English or Spanish. We obtained written informed consent from participants before enrollment according to procedures approved by the University of California, Berkeley, Committee for the Protection of Human Subjects. The serum specimens analyzed in the current investigation were collected between September 1999 and January 2001.

Women were interviewed twice during pregnancy at approximately 13 and 26 weeks gestational age to ascertain information on demographic characteristics, lifestyle habits, work history, and medical history. We also administered a food frequency questionnaire to each participant during the second interview. Blood was collected in the third trimester by venipuncture and immediately processed in a clinical laboratory and frozen at −80°C. Additional information about blood sample collection and processing is presented by Eskenazi et al. (2003).

We measured seven PBDE congeners, including 2,2′,4,4′-tetrabromodiphenyl ether (BDE-47), 2,2′,3,4,4′-pentaBDE (BDE-85), 2,2′,4,4′,5-pentaBDE (BDE-99), 2,2′,4,4′,6-pentaBDE (BDE-100), 2,2′,4,4′,5,5′-hexaBDE (BDE-153), 2,2′,4,4′,5,6′-hexaBDE (BDE-154), 2,2′,3,4,4′,5′,6-heptaBDE (BDE-183), as well as 2,2′,4,4′,5,5′-hexachlorobiphenyl [polychlorinated biphenyl (PCB)-153], and 2,2′,4,4′,5,5′-hexabromobiphenyl [polybrominated biphenyl (PBB)-153] in plasma, and concentrations of target analytes were expressed as nanograms per gram lipid (weight of lipids). The plasma lipid concentration was determined enzymatically using commercially available test kits from Roche Diagnostics Corp. (Indianapolis, IN). For PBDE, PCB-153, and PBB-153 analysis, we extracted the samples according to Hovander et al. (2000). In brief, the samples (0.6–3.6 g) were fortified with ^13^C-labeled internal standards [750 pg/congener, except 2,2′,3,4,4′-pentaBDE (BDE-85), for which no ^13^C-labeled standard existed (Cambridge Isotope Laboratory, Andover, MA)]. The samples were thereafter denaturated with 6 mol/L hydrochloric acid (1 mL) and 2-propanol (6 mL) with mixing between each addition and extracted with *n*-hexane/methyl-*tert*-butyl ether (6 mL, 1:1 by volume) The organic layer was isolated and the water phase reextracted with additional solvent. The extracts were then partitioned against potassium chloride solution (1% by weight in water). The organic fraction was then evaporated to dryness. Co-extracted lipids were removed on a silica/silica:sulfuric acid column packed in a 3-mL solid phase extraction cartridge (Sjödin et al. 2004a). We performed final analytical determination of the target analytes using gas chromatography isotope dilution high resolution mass spectrometry with a MAT95XP (Thermo Electron, Bremen, Germany) instrument (Sjödin et al. 2004a). Three blank and three quality control (QC) samples (unfortified human serum) were included in every set of 24 unknowns (Sjödin et al. 2004a). The limit of detection (LOD) was defined as three times the standard deviation of the blank samples analyzed in parallel with the unknowns; in the absence of a signal in the blank samples, the LOD was defined as 10 times the signal-to-noise ratio. Because of the variable sample size available (0.6–3.6 g), the calculated LOD depended on the sample size, and ranged from 0.1 to 1 ng/g lipid. For all statistical handling of the analytical data, all measurements below the LOD were divided by the square root of two.

The coefficient of variance (CV) for 60 QC samples analyzed during a 9-month period before and after the study specimens reported on in this article were BDE-47 (3%); BDE-85 (10%), BDE-99 (4%), BDE-100 (4%), BDE-153 (5%), BDE-154 (6%), BDE-183 (22%), and PCB-153 (6%) (Sjödin et al. 2004a).

Statistical analysis of the PBDE measurements included computation of descriptive statistics for individual congeners as well as a measure of total exposure to PBDEs (sum of all congeners—BDE-47, BDE-85, BDE-99, BDE-100, BDE-153, BDE-154, and BDE-183). We calculated Spearman correlation coefficients between individual congeners and between PBDE levels and years participants had lived in the United States. Because BDE-99 and BDE-47 are the most commonly detected PBDEs with the highest concentrations, we further evaluated the ratio of BDE-99 to BDE-47 to determine whether this ratio changed over time. Temporal changes in this ratio can provide insight about exposure patterns because their ratio in the environment is known and they have different half-lives in humans. Finally, we used the Fischer’s exact test of the proportion above and below the population median to compare levels by demographic characteristics (STATA Median Test; StataCorp 2001). All statistical analyses were conducted using STATA (StataCorp 2001).

## Results

The levels and distributions for the seven PBDE congeners are presented in Table 1. The total PBDE levels ranged from 5.3 to 320 ng/g lipid, with BDE-47 as the dominant congener. All levels for BDE-47, BDE-99, BDE-100, and BDE-153 were above the LOD. All the PBDE congeners were moderately to strongly correlated with each other (Spearman rho = 0.53–0.95, *p* < 0.05) whereas no significant correlation between PCB-153 and PBB-153 and the PBDE congeners were observed. BDE-183 was excluded from these correlation calculations because > 75% of the measurements were below the LOD (Table 2). The concentration of the sum of PBDEs in two participants exceeded 100 ng/g lipid (8% of the subjects), a proportion similar to that of other studies. (Morland et al. 2005; Schecter et al. 2005).

Table 3 shows selected demographic characteristics of the subjects. Women were on average (± SD) 26 ± 4.8 years of age, overweight, and multiparous with a previous history of lactation. All women were of Mexican descent. Twenty-three were born in Mexico and one in the United States, with more than half having lived in the United States < 5 years (average 5.7 ± 6.9 years). More than half were field workers, and an additional 17% worked in other areas of agriculture.

**Figure 1:**
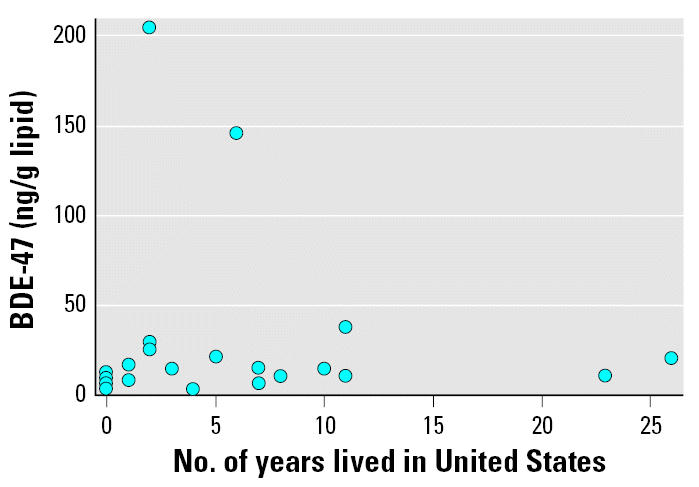
Scatterplot of BDE-47 concentration (ng/g lipid) by years resided in the United States (*n* = 24). BDE-47 and number of years lived in the United States were not significantly correlated.

Few of the demographic characteristics were associated with total PBDE levels or with levels of BDE-47. Overall, levels of PBDE were slightly higher, albeit nonsignificantly, in those women who were agricultural workers, who had a higher body mass index (BMI), and, paradoxically, among parous women with more months of previous lacation (total PBDEs only). We also observed lower levels of PBDEs, in women who consumed more than one serving of fish per month or > 113 g fat/day as compared to women who consumed less. The small sample size limits the interpretation of these comparisons. The correlations of total PBDE and BDE-47 with number of years living in the United States were not significant at a cut-off of *p* < 0.05 (Spearman rho = 0.35, *p* = 0.08; and rho = 0.38, *p* = 0.06, respectively). After excluding the two highest values for participants living in the United States for 2 and 6 years, respectively, the correlations decreased < 0.1 with *p* > 0.5 (Figure 1). BDE-100 and BDE-153 were moderately correlated with years in the United States (Spearman rho = 0.49 and 0.44, respectively) (Table 2). Overall, most Spearman correlations were positive, suggesting that levels increased with time in the United States and might have been significant had the sample size been larger. Levels of PCB-153 (median = 4.4 ng/g lipid, range, < 2–75) were lower than U.S. averages and uncorrelated with PBDE levels (Table 2).

**Figure 2:**
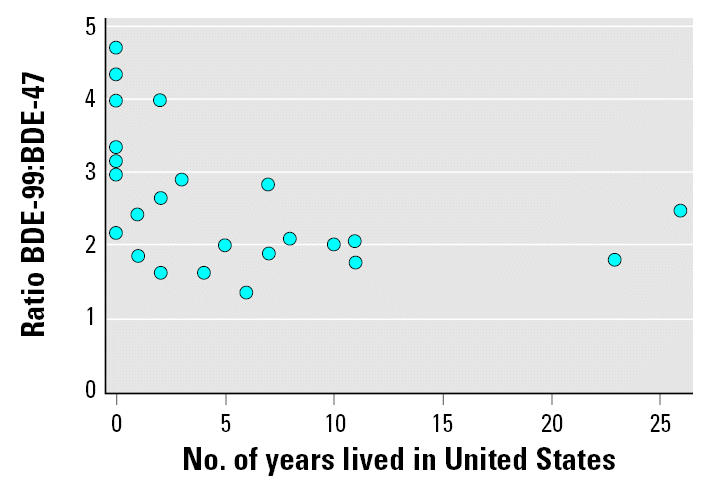
Scatterplot of the ratio of BDE-99 to BDE-47 by years resided in the United States (*n* = 24). The ratios are highest among women who had spent < 5 years in the United States compared with women who had spent > 5 years in the United States (Spearman rho = −0.59, *p* < 0.01).

The median ratio of BDE-99 to BDE-47 in our sample was 0.23, with a maximum of 0.47. Figure 2 presents a scatterplot of the ratio of BDE-99 to BDE-47 by the number of years the women had been in the United States. The ratios are highest among women who have spent < 5 years in the United States compared to women who have spent > 5 years in the United States (Spearman rho = −0.59, *p* < 0.01).

## Discussion

This pilot study indicates that women of Mexican origin living in a California agricultural community were exposed to PBDEs. The small sample size limits our ability to determine which factors are associated with these exposures. Preliminary analyses suggest that there were no clear associations of demographic characteristics including age, lactation, and parity with blood levels of PBDEs. The data suggest that PBDE levels are somewhat higher in women with higher body mass, who had lower fish consumption, who worked in agriculture, and, paradoxically, among parous women with more months of previous lacation (total PBDEs only).

Only one previous study in the United States was conducted in an immigrant population (Petreas et al. 2003). In that study of women from Southeast Asia, only BDE-47 was measured, and 52% of the women’s BDE-47 levels were not detectable due to high detection limits. The median BDE-47 level in the South Asian women was 10 ng/g lipid (range, < 10–511) and comparable to our cohort of Mexican immigrants having a median BDE-47 level of 11 ng/g lipid (range, 2.5–205) but lower than that reported for a cohort of women from California (median = 18 ng/g lipid; range, 7.01–196) (She et al. 2002). Similar to our findings, Petreas and coauthors (2003) did not find a positive association between PBDE levels and age. This lack of an increase with age is in contrast to other persistent organic pollutants and has been previously reported for PBDEs (Darnerud et al. 2001).

The median BDE-99/BDE-47 ratio of 0.23 in our data is consistent with that of previous human studies (Morland et al. 2005; Schecter et al. 2005; She et al. 2002) and reflects a normal metabolized congener pattern that is different from the pattern found in indoor dust and technical pentaBDE (a ratio generally greater than one). The lower level of BDE-99 compared to BDE-47 in biologic samples could be caused by the shorter half-life of BDE-99 relative to BDE-47 and also suggests that no contamination of our samples by exogenous PBDEs, which have a higher ratio (Sjödin et al. 1998, 2005), occurred during sample collection and handling. We also report that the ratio of BDE-99 to BDE-47 was higher in women who recently immigrated to the United States (Figure 2). We have no ready explanation for this finding. One possibility is that the women may have experienced lower exposures to PBDEs from indoor dust in their home country than in the United States. At least compared to Europe, house dust in the United States contains high levels of PBDE congeners found in commercial pentaBDE, including BDE-99 and BDE-47 in ratios close to unity. For example, the indoor dust concentration of BDE-47 in Germany has been reported to be < 14–22 ng/g versus 230–3,000 ng/g reported in the United States (Sjödin et al. 2005). No data are available on PBDE levels in residential environments in Mexico; however, overall use is negligible compared to the United States (Hale et al. 2003), suggesting that residential environmental contamination is likely to be low. Thus, on arrival in the United States, the women likely experienced exposures to dust with a higher PBDE concentration. Because BDE-99 is less persistent in humans, the ratio in our participants possibly decreased over time as BDE-99 was excreted and BDE-47 was retained. It is also possible that continued exposure from other environmental or dietary sources and bioaccumulation of BDE-47 altered the relative balance of these congeners over time.

The high degree of correlation between the different PBDE congeners analyzed and the complete absence of correlation between the PBDE congeners and PCB-153 (Table 2) may suggest that the exposure routes for these two classes of compounds are different. It is well known that humans are exposed to PCBs to a large extent though dietary sources (Patandin et al. 1999). Hence, the absence of correlation between PCB-153 and the PBDEs may indicate that nondietary pathways are of greater importance for PBDEs, a finding consistent with data recently reported by Schecter et al. (2006).

Our study provides data on PBDE levels in a Mexican population living in a U.S. agricultural community. The overall levels of PBDE found in this study were slightly lower than levels observed in other U.S. populations, although the upper range of exposure is similar. Nevertheless, these levels were higher than those observed previously in Europe or Japan (Hites 2004). As in previous U.S. studies (Hites 2004), we found that the congener with the highest levels was BDE-47. PBDEs have been shown to cause adverse developmental effects in animal experiments (Eriksson et al. 2001; McDonald 2005; Viberg et al. 2003). Future studies should determine the sources and pathways of PBDE exposures and whether these exposures have adverse impacts on human neurodevelopment.

## Figures and Tables

**Table 1:** Concentration (ng/g lipid) of measured PBDEs, PBBs, and PCBs in selected samples (*n* = 24) from a cohort of pregnant Latina women in California.

			Selected Percentiles^a^				
Compound	Detection frequency (%)	LOD^b^	Minimum	25th	50th	75th	Maximum
PBDEs							
BDE-47	100	—	2.5	7.6	11	21	205
BDE-85	71	0.2–1	< 0.1^b^	< 0.1^b^	0.3	0.5	5.0
BDE-99	100	—	0.5	1.9	2.9	4.5	54
BDE-100	100	—	0.6	1.2	1.8	3.2	44
BDE-153	100	—	0.4	0.9	1.5	2.8	35
BDE-154	75	0.2–0.5	< 0.1^b^	< 0.2^b^	0.3	0.4	4.2
BDE-183	33	0.1–0.5	< 0.1^b^	< 0.1^b^	< 0.1^b^	0.4	39
Sum of PBDEs	100	—	5.3	13	21	34	320
PCBs and PBBs							
PCB-153	96	2	< 2^b^	2.8	4.4	6.6	75
PBB-153	38	0.2–1	0.1	0.2	0.2	0.4	1.5

a Percentiles calculated based on results for all (*n* = 24) samples.

b The calculated LOD varied by sample due to variable sample sizes (0.6–3.6 g); for all statistical analyses, all measurements below the LOD were divided by the square root of two.

**Table 2:** Spearman’s rank-order correlation coefficients between individual PBDE congeners and PCB-153.

Compound	BDE-47	BDE-85	BDE-99	BDE-100	BDE-153	BDE-154
BDE-85	0.91**					
BDE-99	0.92**	0.92**				
BDE-100	0.95**	0.88**	0.86**			
BDE-153	0.6**	0.62**	0.53**	0.75**		
BDE-154	0.58**	0.69**	0.66**	0.65**	0.77**	
PCB-153	0.13	0.17	0.16	0.15	0.09	0.06
Years in United States	0.35	0.19	0.19	0.49*	0.44*	0.25

* *p* < 0.05;

** *p* < 0.01.

**Table 3:** Demographic characteristics of study participants (*n* = 24) and relationship with BDE-47 and total PBDEs (ng/g lipids).

Characteristic	No. (%)	BDE-47 (median)	Total PBDEs (median)
Age (years)			
≤ 25	12 (50)	11.3	20.8
> 25	12 (50)	12.2	20.4
Years in United States			
< 5	14 (58)	10.0	18.7
≥ 5	10 (42)	14.5	22.9
Parity			
0	10 (42)	11.3	18.7
≥ 1	14 (58)	12.2	22.6
Previous lactation (months, parous women only)			
< 12	6 (43)	12.2	19.4
≥ 12	8 (57)	12.5	27.7
Agricultural work			
None	6 (26)	7.1	14.2
Farm	17 (74)	14.4	23.0
BMI			
Under/normal	10 (42)	10.7	18.7
Over/obese	14 (58)	14.3	22.6
Fish consumption (servings)			
< 1/month	14 (61)	14.7	22.6
≥ 1/month	9 (39)	10.1	17.8
Dairy consumption (servings)			
< 2.3/day	12 (50)	11.3	18.7
≥ 2.3/day	12 (50)	12.3	22.6
Total fat (g)			
< 113/day	12 (50)	12.8	21.3
≥ 113/day	12 (50)	9.5	20.0
